# Degradation of Diclofenac by Loaded Solid Superbase-Activated Persulfate

**DOI:** 10.3390/ijms241814313

**Published:** 2023-09-20

**Authors:** Jiaqi Shi, Lei Wang, Shang Gao, Jianbo Huang, Hao Yang, Hao Lu, Shaohua Cao

**Affiliations:** 1 State Environmental Protection Key Laboratory of Soil Environmental Management and Pollution Control, Nanjing Institute of Environmental Sciences, Ministry of Ecology and Environment of China, Nanjing 210042, China; sjq@nies.org (J.S.); leiwang@nies.org (L.W.); gaoshang@nies.org (S.G.); hjb@nies.org (J.H.); 15215658304@163.com (H.L.); 2 State Key Laboratory of Pollution Control and Resource Reuse, School of the Environment, Nanjing University, Nanjing 210023, China; 3 College of Environment, Hohai University, Nanjing 210098, China; 4 College of Resources and Environmental Engineering, Hefei University of Technology, Hefei 230009, China

**Keywords:** base-activated persulfate, catalytic mechanism, diclofenac, solid superbase

## Abstract

Alkali-activated persulfate (PS) is widely used in situ in chemical oxidation processes; however, studies on the innovation of the alkali activation process are very limited. Two supported solid superbases, namely KNO_3_/γ-Al_2_O_3_ (KAl) and KNO_3_/SBA-15/MgO (KSM), respectively, were prepared and used to activate persulfate to degrade DCF in this work. The results showed that the superbases elevated the solution pH once added and thus could catalyze persulfate to degrade diclofenac efficiently above pH 10.5. The catalytic efficiency of KAl was close to that of sodium hydroxide, and that of KSM was the highest. The mechanism might be that, in addition to raising the solution pH, some potassium existed as K_2_O_2_, which had a strong oxidizing effect and was conducive to DCF removal. Hydroxyl, sulfate and superoxide radicals were all found in the reaction system, among which hydroxyl might play the most important role. The material composition ratio, common anion and humic acid all had some influences on the catalytic efficiency. A total of five intermediates were found in the KSM/PS oxidation system, and six oxidation pathways, which were hydroxylation, dehydrogen, dechlorination, dehydration, decarboxylation, and C-N bond breakage, might be involved in the reaction process. Several highly toxic oxidation products that should be paid attention to were also proposed.

## 1. Introduction

Diclofenac (DCF) is a typical non-steroidal anti-inflammatory that is widely used in clinical practice to treat painful rheumatoid and non-rheumatoid diseases and is one of the most frequently detected pharmaceutical compounds in the environment, such as wastewater, surface water, soil and groundwater [1,2]. With the global outbreak of COVID-19, a large number of such pharmaceuticals might accumulate in the environment furthermore [3,4]. DCF poses a serious threat to the ecological environment and human health and is resistant to biodegradation in the activated sludge process [2,5,6]. The advanced oxidation process (AOP) is an important method to remove DCF; however, previous studies have shown that the toxicity of DCF solution experienced an increased stage during some AOP processes and that the toxicity might be related to a generation of chloro derivatives [7,8,9].

Activated persulfate (PS) oxidation has been widely used in pollutant removal. It uses heat, transition metal, UV and alkali as the activator to produce sulfate radicals (•SO_4_^−^) and degrade pollutants [10]. Among the activation methods, alkali activation is one of the most popular in situ chemical oxidation processes. It has been revealed that reducing radicals such as superoxide radical (•O_2_^−^) could be produced in the alkali-activated persulfate system and facilitate the dechlorination of chlorinated alkanes and desorption of contaminants in the soil system [11]. However, studies on the innovation of the alkali activation process and its mechanisms are still limited, which might be related to its low catalytic efficiency. Solid superbase is a novel catalyst used for the chemical synthesis of pharmaceuticals, biodiesel, sucrose esters and other products. It has received much attention in the disciplines of catalysis because of its stronger catalytic activity, better selectivity, regenerability, lower ecological pollution and less corrosiveness to reaction equipment compared with conventional strong bases [12,13]. As the solid superbase has a higher alkali strength than conventional strong bases, it is supposed to have the potential to activate persulfate to degrade organic pollutants. In addition, solid superbases could overcome the strong corrosive effect of conventional bases on metal pipelines [14,15]. As a result, this work aims to investigate the application feasibility of a solid superbase in environmental remediation. The γ-Al_2_O_3_, modified by alkali metals, and the mesoporous materials, modified by alkali metals, are two typical types of superbase materials [13], and as a result, KNO_3_/γ-Al_2_O_3_ (KAl) and KNO_3_/SBA-15/MgO ((*y*)KSM(*x*)) were prepared and used to activate persulfate to degrade DCF. In (*y*)KSM(*x*), *y* represented the mass percent of KNO_3_, and *x* represented the mass percent of MgO of the total mass. The catalytic efficiency, influencing factors, oxidation mechanism and product toxicity will be revealed in this work.

## 2. Results and Discussion

### 2.1. Identification of Free Radical Species

**Figure 1:**
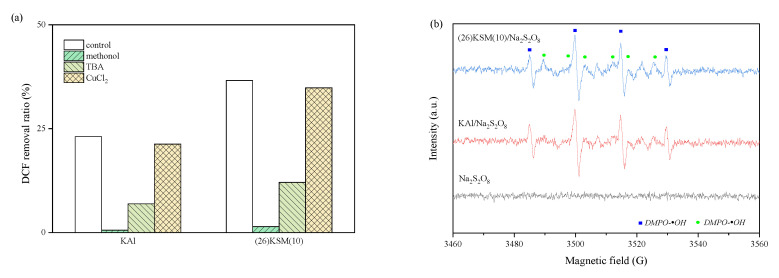

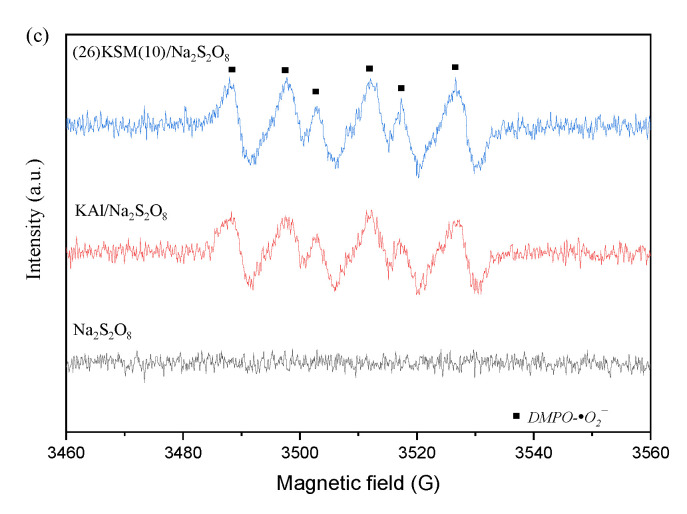
(**a**) Effect of quenching agents on DCF degradation; (**b**) aqueous dispersion for DMPO-•OH and DMPO-•SO_4_^−^; (**c**) methanol dispersion for DMPO-•O_2_^−^.

The 4 h removal ratio of DCF with different quenching agents in the KAl/PS and (26)KSM(10)/PS systems is shown in Figure 1a. It can be seen that the inhibition effects were similar in the two systems. DCF removal was almost completely suppressed when methanol existed in the solution, indicating that free radicals played dominant roles in the two oxidative systems. The tert-butanol (TBA) reduced the DCF removal ratio by 16.2% and 24.5%, respectively, which indicated that •OH played a more important role than •SO_4_^−^. The DCF removal ratios after a CuCl_2_ addition were 21.3% and 34.8%, respectively, which were slightly lower than those of the control (23.1% and 36.6%), indicating that •O_2_^−^ might exist in the systems.

The quenching and electron paramagnetic resonance (EPR) spectra proved the existence of •OH, •SO_4_^−^ and •O_2_^−^. It can be seen from Figure 1b that the typical addition products, DMPO-•SO_4_^−^ and DMPO-•OH, were produced in the persulfate oxidation system. The peak intensity in the (26)KSM(10)/PS group was slightly higher than that in KAl/PS. The response of •OH was much stronger than that of •SO_4_^−^, meaning that •OH played a major role, which was consistent with the result from the quenching test. The •O_2_^−^ was also found to have a strong response from the EPR result (Figure 1c).

**Figure 2:**
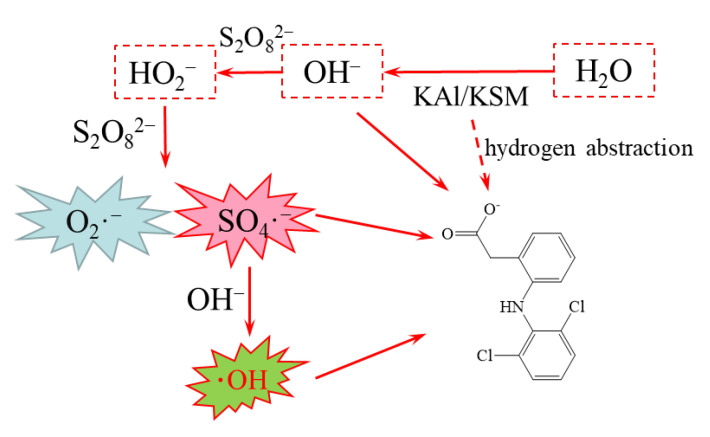
The proposed mechanism of loaded solid superbase-activated persulfate to oxidize pollutants.

Based on the analysis of free radicals, it is speculated that the activating mechanism by the solid superbase was that the strong proton acceptance capacity of the superbase brought the rapid increase of pH. The OH^−^ activated the PS to produce •SO_4_^−^ and •O_2_^−^, and then •SO_4_^−^ reacted with OH^−^ to yield •OH, which was similar to that of the base-activated persulfate [11]. As for some contaminants with high acidity constants, superbases may also directly deprotonate them, making them more susceptible to oxidation, especially in the non-aqueous-phase liquid (NAPL) phase [13,16]. The presumed activation mechanism of the bases is shown in Figure 2.

### 2.2. DCF Removal Effect by Different Bases Activated Persulfate

**Figure 3:**
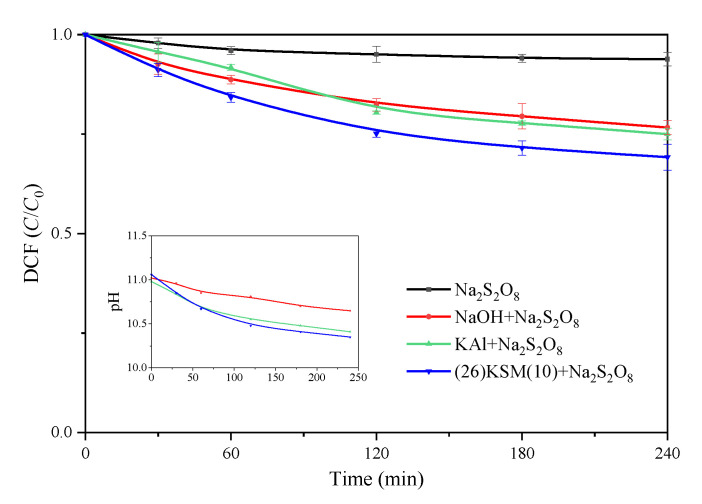
DCF removal in different base-activated persulfate systems. The data points represent the average values of the parallel tests, and the error bars are the standard deviations.

When 0.1 g of KAl or (26)KSM(10) was added to the 25.0 mL reaction solution, the initial pH values were 10.98 and 11.06, respectively. The pH of the NaOH/PS system was adjusted to 11.02. The DCF removal results are shown in Figure 3. It can be seen that all the base-activated persulfate systems had stronger oxidizing abilities than persulfate itself. DCF was removed in a similar ratio in the KAl and NaOH groups, while the degradation efficiency was significantly higher in the (26)KSM(10)/PS group after 4 h. The degradation reactions in the base-activated oxidation systems followed pseudo-first-order kinetics in the entire 4 h period.

The pH experienced decreased trends in all the base-activated persulfate groups, among which the pH in the (26)KSM(10)/PS system decreased most obviously, and in the NaOH/PS group, it decreased most gently. The results were consistent with the removal trend of DCF. The higher the catalytic efficiency, the more significant the pH reduction. This indicated that the degradation efficiency did not only depend on the pH; however, when the pH decreased to about 10.5, the oxidation rates were inhibited in all groups. As potassium oxide (K_2_O) decomposes to potassium peroxide (K_2_O_2_) and potassium metal at high temperatures [17], the fact that the superbases had a higher activate ability might be because the loaded potassium did not only exist in the form of K_2_O and released into solution to improve pH, but some existed as K_2_O_2_ and was conducive to the oxidation of pollutants.

### 2.3. XPS Analysis

**Figure 4:**
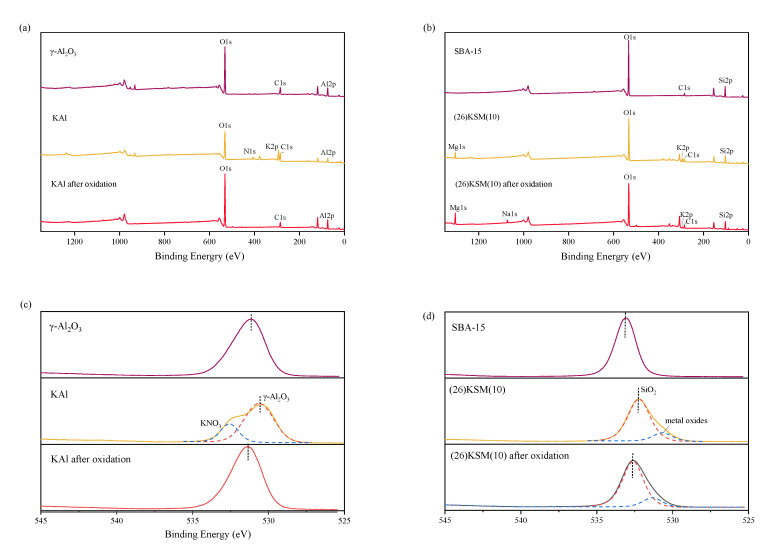
XPS survey spectrum of (**a**) KAl and (**b**) (26)KSM(10), and O1s spectra of (**c**) KAl and (**d**) (26)KSM(10).

In order to verify the alkali adsorption and residue on the superbase materials, the X-ray photoelectron spectroscopy (XPS) analysis, before and after oxidation, was carried out. As shown in Figure 4a, potassium was loaded onto KAl successfully after baking, and nitrogen was also introduced, indicating that a partial potassium element might still exist in the form of KNO_3_, resulting in a slightly lower initial pH for the KAl/PS system than the (26)KSM(10)/PS system. After catalytic oxidation for 24 h, the potassium and nitrogen were both removed from the material, indicating that the alkaline had been eliminated. It can be seen in Figure 4b that KNO_3_ has completely decomposed into K_2_O in (26)KSM(10). After catalytic oxidation for 24 h, the potassium still existed at quite a low dose, and sodium from PS was adsorbed onto the material. Figure 4c,d describe the O1s binding energy of the two solid bases. Previous studies have shown that a lower O1s binding energy indicates a stronger electron donating ability, meaning a higher alkali strength [18]. The O1s binding energies of the KAl and (26)KSM(10) materials were lower than those of the parent material, which proved that alkali was loaded into the materials. The O1s spectra of KAl could be deconvoluted into two peaks at 530.7 eV and 532.5 eV. The peak at 530.7 eV corresponded to alkaline-enhanced γ-Al_2_O_3_, and that at 532.5 eV corresponded to KNO_3_. The characteristic peaks of O1s were fitted by two peaks in (26)KSM(10) at 530.6 eV and 532.2 eV, corresponding to metal oxides and alkaline-enhanced SiO_2_, respectively. The O1s spectra proved that the alkalinity of the two materials was weakened severely after 24 h of catalytic oxidation. KAl lost its alkalinity totally, while (26)KSM(10) lost most of its alkalinity; however, the alkalinity was still stronger than SBA-15, and a small amount of alkalinity remained on the solid’s surface.

### 2.4. Influence of MgO and Alkali Loading Ratio on KSM-Activating Effect

**Figure 5:**
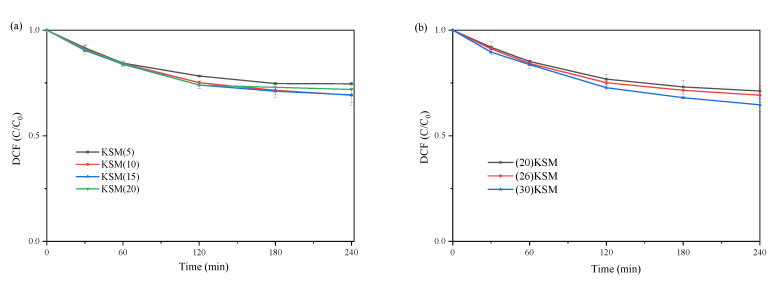
DCF degradation by persulfate oxidation systems with different loading ratios of (**a**) MgO and (**b**) KNO_3_.

The load proportions of MgO in KSM were set to 5%, 10%, 15% and 20%, respectively. Figure 5a shows the catalytic degradation kinetics of DCF under different MgO loading ratios. It can be seen in Figure 5a that the load proportion of MgO had a slight influence on the DCF removal efficiency. The solid superbases loaded with 10 wt% and 15 wt% MgO have the highest efficiency in activating persulfate. Wu et al. [19] revealed two reasons for covering SBA-15 with MgO. One reason was that the coated MgO entered the micropores of SBA-15, forming a protective layer, which would prevent the silica skeleton from being corroded by potassium. The other was that MgO coated on SBA-15 could promote the decomposition of KNO_3_ to produce the alkaline substance, K_2_O, under high-temperature treatment. It was found that the introduction of 1~9 wt% MgO to SBA-15 did not completely protect the silica matrix of SBA-15, and the structure of SBA-15 was almost completely preserved only when the in situ coated MgO content reached 10 wt% or more. However, too much MgO would affect the specific surface area and pore space of the material, thus affecting the dispersion of KNO_3_. The above studies could explain the degradation results of DCF in this study.

Figure 5b depicts the effect of different amounts of KNO_3_ loading onto SBA-15 coated with 10% MgO on the degradation of DCF by activating persulfate. After 4 h of reaction, it can be demonstrated that the material loaded with 26 wt% and 30 wt% KNO_3_ is slightly more effective than that loaded with 20 wt% KNO_3_ in activating persulfate to remove DCF. The reason may be that the higher loading ratio of KNO_3_ indicated a stronger alkalinity. Therefore, it has higher catalytic efficiency when the MgO loading ratio is between 10% and 15% and the KNO_3_ loading ratio is above 25%.

### 2.5. The Effect of Inorganic Anions and Humic Acid

**Figure 6:**
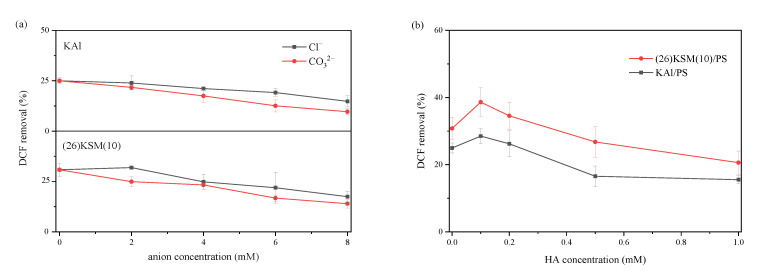
Effects of Cl^−^, CO_3_^2−^ and humic acid on the degradation efficiency of DCF in KAl/PS and (26)KSM(10)/PS systems: (**a**) shows the effect of Cl^−^ and CO_3_^2−^, and (**b**) shows the effect of humic acid.

The influence of Cl^−^, CO_3_^2−^ and humic acid—which are prevalent in the environment—on DCF degradation was investigated. It can be seen in Figure 6a that the 4 h degradation ratio of DCF decreased continuously as the concentration of Cl^−^ or CO_3_^2−^ increased in the range of 1–8 mM, and the inhibitory effect of CO_3_^2−^ was greater than that of Cl^−^ in both the KAl/PS and (26)KSM(10)/PS systems. When the solution contained 8 mM Cl^−^, the degradation ratios of DCF were inhibited by 10.2% and 13.4%, respectively, in the KAl/PS and (26)KSM(10)/PS systems, and were inhibited by 15.3% and 16.8%, respectively, in the CO_3_^2−^ cases. The main reason for the inhibition of the reaction by anions was that Cl^−^ and CO_3_^2−^ consumed •OH and •SO_4_^−^, as shown in Equations (1)–(4) [20,21].

CO_3_^2−^ + •SO_4_^−^ → •CO_3_^−^ + SO_4_^2−^ (1)

CO_3_^2−^ + •OH → •CO_3_^−^ + OH^−^ (2)

Cl^−^ + •SO_4_^−^ → •Cl + SO_4_^2−^ (3)

Cl^−^ + •OH → •Cl + OH^−^ (4)

The 0.1 mM humic acid seemed to promote DCF removal; however, a higher concentration inhibited it significantly. The possible reason was that the structures, such as the phenolic hydroxyl, carbonyl and carboxyl groups, contained in humic acid molecules, activated persulfate to generate oxidation radicals [22,23]. Shi et al. also found that the quinone structure catalyzed persulfate most efficiently around pH 10.0, as the hydroquinone in anionic form could activate persulfate efficiently, as shown in Equations (5) and (6) [24]. The competition with the target pollutant became the dominant effect when the concentration was further increased, as the •OH was non-selective and easily consumed by HA.

C_6_H_5_O_2_^−^ + S_2_O_8_^2−^ → •SO_4_^−^ + SO_4_^2−^ + C_6_H_5_O_2_• (5)

C_6_H_5_O_2_• + S_2_O_8_^2−^ → •SO_4_^−^ + C_6_H_4_O_2_ (6)

### 2.6. Proposed Degradation Pathways and Toxicity Assessment of Products

**Figure 7:**
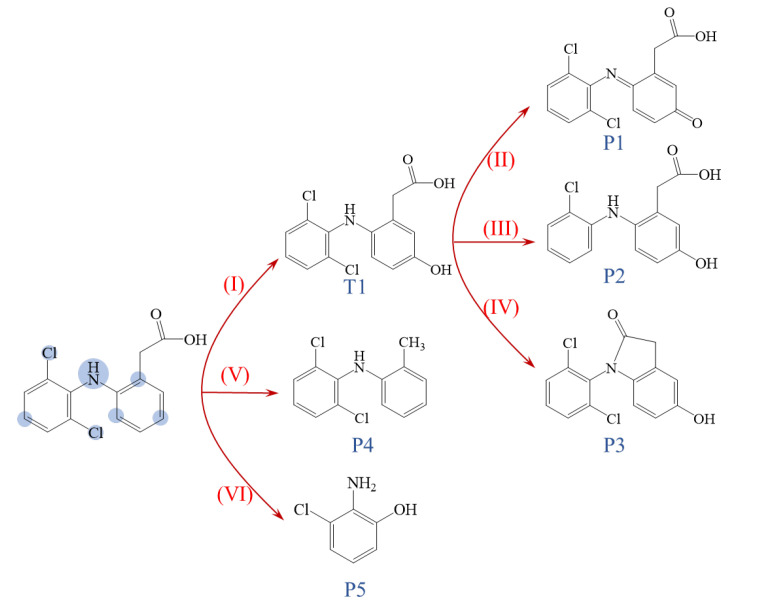
The proposed oxidation pathway in the KSM/PS system. The numbers indicated the DCF degradation pathways.

A total of five intermediates were found in the KSM/PS oxidation system. The chromatogram is shown in Figure S4 of the Supplementary Information (SI). The degradation pathway of DCF was speculated based on the structure of the degradation intermediates, as shown in Figure 7. Six oxidation pathways may be involved in the reaction process. Pathway I was hydroxylation, which provided 5-hydroxydiclofenac (T1) and then, T1 was further dehydrogenated (pathway II) to be quinone (P1). Pathway III was dechlorination, which produced P2 from T1. Dehydration (pathway IV) also occurred to form P3. The carboxyl might also be removed to produce P4 (pathway V), and breakage of the C-N bond (pathway VI) produced 2-amino-3-chlorophenol (P5). The proposed oxidation pathways were similar to previous studies [7,25].

In a solid superbase-activated persulfate system, the hydroxyl radicals play a dominant role and have a strong electron-gaining ability, and therefore, are likely to attack those sites that are susceptible to electron loss first. Chen et al. calculated the Fukui index distribution of radical attacks (*f*^0^) on the DCF molecule [26], and the atoms that are susceptible to attacks are shown in Figure 7. The oxidation pathways correspond to the distribution of *f*^0^ quite well. The transformation usually occurs at the positions susceptible to radical attack.

### 2.7. Toxicity Assessments

**Figure 8:**
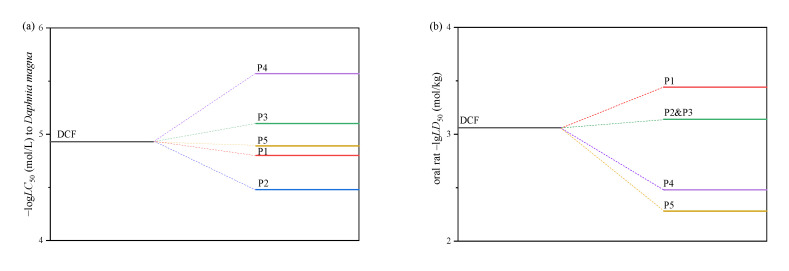
Acute toxicity of DCF and the main oxidation products. (**a**) 48 h *Daphnia magna* log*LC*_50_ (mol/L), (**b**) oral rat –lg*LD*_50_ (mol/kg).

According to previous research, the oxidation of DCF resulted in a rise in toxicity that was associated with products that were more toxic than DCF itself [7]. However, it is still unknown which products resulted in the toxicological increase [27]. Therefore, it is necessary to assess the toxicity variation of DCF during the superbase/PS process. The calculated toxicity results are shown in Figure 8. It is seen that P3 and P4 have a stronger acute toxicity to *Daphnia magna,* while P1, P2 and P3 have a stronger oral acute toxicity to rats. Due to the low responses, the overall toxicity would not be affected significantly; however, the accumulation of highly toxic products should be paid attention.

## 3. Materials and Methods

### 3.1. Chemical Reagents and Equipment

Diclofenac sodium was provided by Sigma-Aldrich (St. Louis, MO, USA), and potassium nitrate, copper chloride (CuCl_2_) and PS were obtained from Sinopharm Co., Ltd., Shanghai, China. Magnesium oxide (MgO), γ-aluminium oxide (γ-Al_2_O_3_), humic acid (fulvic acid ≥ 90%), HPLC-grade methanol and TBA were all obtained from Shanghai Aladdin Biochemical Technology Co., Ltd., Shanghai, China. SBA-15 was obtained from XFNANO Co., Ltd., Nanjing, China. The theoretical molecular formula of humic acid was C9H9NO6, and it is calculated in terms of 227 g/mol when calculating the molar concentration.

### 3.2. Preparation of Solid Superbases

The KAl and (*y*)KSM(*x*) superbases were prepared by referring to the published procedures [19,28]. Briefly, to prepare KAl, the 26 wt% of KNO_3_ and 74 wt% of γ-Al_2_O_3_ were mixed. Then, some distilled water was added, and the mixture was ground into a paste. After drying it at 110 °C for 2 h, the sample was baked at 600 °C for 5 h in nitrogen. SBA-15 was coated with the *x* wt% of MgO and *y* wt% of KNO_3_, respectively. The (26)KSM(10) sample was used in most of the cases in this work. After the sample was ground to a paste, it was baked at 600 °C for 5 h under nitrogen. The materials were stored in a vacuum drier before use.

### 3.3. Characterization of Solid Superbase

The surface morphology of the materials was characterized by N_2_ adsorption at 77.35 K using a gas adsorption instrument (Autosorb-iQ, Quantachrome, Boynton Beach, FL, USA). Each catalyst was treated at 250 °C for 4 h to remove impurities before measurement. The total surface area and mesoporous surface area were measured using the Brunauer–Emmett–Teller (BET) and Barrett–Joyner–Halenda (BJH) methods, respectively. The total pore volume was calculated from the amount of nitrogen absorbed at a P/P0 of 0.99. The pore size distribution was drawn from the desorption branch with the BJH method.

The CO_2_-temperature programmed desorption (CO_2_-TPD) reflected the generation of strong basic sites in the samples. The test was performed with an Autosorb-iQ-C chemisorption analyzer (Quantachrome, Boynton Beach, FL, USA). Before the TPD experiments, the 50 mg material was heated in a He stream at a rate of 13 °C·min^−1^ to 800 °C and remained for one hour. The sample was subsequently cooled down to room temperature and saturated with pure CO_2_. After purging with a He stream at 25 °C for 1 h to remove non-specifically physiosorbed CO_2_, the desorbed CO_2_ was measured by heating the sample from 25 °C to 800 °C at a rate of 13 °C·min^−1^. The CO_2_-TPD profiles of KAl and (26)KSM(10) are shown in Figure S3 of the SI.

XPS analysis was performed to observe the elemental species and O1s binding energies on an ESCALAB 250xi (Thermo Scientific, Waltham, MA, USA). XPS used an Al K_α_ radiation source (1486.4 eV) at room temperature and under a vacuum pressure of 10^−7^ Pa.

The surface characteristics of KAl and (26)KSM(10) analyzed with a scanning electron microscope (SEM) and BET are shown in the Figures S1 and S2 and Table S1, respectively of the SI.

### 3.4. Degradation Experiment

The degradation experiments were carried out in a 50 mL brown glass bottle on a magnetic stirrer apparatus with a rotor stirring in it. The temperature was maintained at about 25 °C, and the rotation speed was set at 200 rpm. The initial concentration of DCF was 5 mg·L^−1^, and 0.1 g of solid superbase material was added. The PS stoke solution was then added to the reaction solution, which reached a final concentration of 15 mM to trigger the reaction. The total reaction volume was 25 mL. An amount of 5 mL of the sample was taken out with a pipette into a 10 mL brown glass tube, and 2 mL of methanol was added to terminate the reaction before the PS addition and after the reaction for 30 min, 1 h, 2 h, 3 h and 4 h, respectively. Then, 1 mL of the sample was filtered through a 0.45 µm aqueous membrane and injected into a 1.5 mL vial for DCF quantification. The degradation experiments were conducted in triplicate, and the results are shown as the mean ± standard deviation (SD), as shown in Figure 3, Figure 5 and Figure 6.

### 3.5. Analytical Methods

The concentrations of DCF were measured by high-performance liquid chromatography (HPLC, Waters 2988) using a Hypersil GOLD C18 column (dimensions 250 mm × 4.6 mm, 5 μm, Thermo) equipped with a UV–Vis detector at a wavelength of 278 nm [29]. The mobile phase consisted of methanol and 1% formic acid (75:25, *v*/*v*) at 1.0 mL·min^−1^ while the column temperature was maintained at 30 °C. The injection volume was 10 μL.

The quenching and EPR experiments were conducted, respectively, to detect and identify the species and intensity of the free radicals in the DCF degradation process. Methanol, TBA and CuCl_2_ were selected as quenching agents to explore the role of different free radical species by comparing the reaction ratios. Methanol reacts at high and comparable rates with hydroxyl radical·(•OH) and sulfate radical (•SO_4_^−^). Its reaction rate with •OH and •SO_4_^−^ were (8.0~10) × 10^8^ M^−1^ s^−1^ and (0.9~1.3) × 10^7^ M^−1^ s^−1^, respectively. The reaction rate of TBA with •OH ((3.8~7.6) × 10^9^ M^−1^ s^−1^) is approximately 1000-fold greater than that with •SO_4_^−^ ((4.0~9.1) × 10^5^ M^−1^ s^−1^) [30,31]. CuCl_2_ is a disproportionation agent of •O_2_^−^ [32].

During the EPR experiment, 200 µL of the reaction solution at 4 h was taken and mixed with 100 µL of trapping agent (5,5-dimethyl-1-pyrroline-N-oxide, DMPO, 100 mM). The mixed aqueous solution was immediately injected into the capillary tube and inserted into the EPR spectrophotometer (Bruker A300, Ettlingen, Germany) for the detection of •OH and •SO_4_^−^. Moreover, DMPO was also used to detect •O_2_^−^ in the methanol solution. The resulting spectrum was compared with the standard spectrum to determine the major radical species.

The oxidation products were analyzed using solid-phase extraction (SPE) followed by an UltiMate 3000 ultrahigh-performance LC (UHPLC) system coupled with a Q Exactive mass spectrometer (Thermo Fisher Scientific, Waltham, MA, USA). The Oasis hydrophilic–lipophilic-balanced (HLB) SPE cartridge was preconditioned with 1 mL of methanol and 1 mL of deionized water, respectively. Then, 5 mL of the solution was taken out after the reaction for 240 min from the KSM/PS system and was acidized with 2% formic acid and loaded into the cartridge (30 mg, 3 mL, Waters, Milford, MA, USA). The cartridge was rinsed with 3 mL of deionized water with 5% methanol (*v*/*v*), dried under vacuum for 2 min, and eluted with 3 mL of methanol at a rate of 3 mL/min. The eluates were concentrated to 1 mL under N_2_ and transferred into an autosampler vial for instrumental analysis. Chromatographic separation was performed on a BEH C18 column (100 × 2.1 mm, 1.7 μm). The mobile phase consisted of (A) acetonitrile and (B) water with 0.1% formic acid (*v*/*v*). The flow rate was 1 mL/min. The solvent gradient was 5% A initially and was increased gradually to 100% A until 15.00 min and maintained for 5 min. The mass analyzer was operated in the full scan mode over a mass range of *m*/*z* 100−600. Electrospray ionization (ESI) conditions, including capillary voltage, capillary temperature, probe heater temperature, and sheath gas flow, were optimized at 3.6 kV (positive ion mode), 350 °C, 320 °C, and 40 arbitrary units, respectively.

### 3.6. Toxicity Prediction

The toxicity of DCF and its oxidative products were evaluated by the US EPA Toxicity Estimation Software Tool (T.E.S.T, Version 5.1.1) with the quantitative structure-activity relationship (QSAR) mathematical models (US EPA, 2022). The 50% lethal concentration (*LC*_50_) to *Daphnia magna* (48 h) was calculated using the consensus method, and 50% lethal doses (*LD*_50_) to the oral rat were calculated using the nearest neighbor method in this work.

## 4. Conclusions

The present study proved that loaded solid superbases elevated the solution pH rapidly and activated persulfate to produce •OH, •SO_4_^−^ and •O_2_^−^, among which •OH might play the most important role according to the quenching and EPR results. The degradation efficiency of diclofenac was a little higher in the superbase/PS solutions than in the NaOH/PS system at a similar pH. The reason may be that the loaded potassium did not only exist in the form of K_2_O and was released into the solution to improve the pH; however, some might exist as K_2_O_2_ and be conducive to the oxidation of pollutants. The composition ratio during the superbase synthesis process might influence the catalytic efficiency. The 1–8 mM Cl^−^ and CO_3_^2−^ both inhibited oxidation; however, humic acid in a low concentration might promote it. DCF could be oxidized through hydroxylation, dehydrogenation, dechlorination, dehydration, decarboxylation, C-N bond breakage, etc. The quinonized, decarboxylated and cyclization products might have the potential to promote toxicity of the solution. This work proved the feasibility of using superbase to activate PS and initially revealed the activation mechanism, influencing elements and the transformation pathway of DCF. However, the optimization methods of superbase materials, applicable scenarios and action mechanisms still need to be further explored.

## Data Availability

The data that support the findings of this study are available within the article and the Supplementary Information. Further data are available from the corresponding author upon reasonable request.

## Supplementary Materials

The supporting information can be downloaded at: https://www.mdpi.com/article/10.3390/ijms241814313/s1. References [33,34] are cited in Supplementary Materials.
