# Supplementary material for: Degradation of Diclofenac by Loaded Solid Superbase-Activated Persulfate
Source: Int J Mol Sci. 2023 Sep 20;24(18):14313. doi: 10.3390/ijms241814313 (PMC10531577; doi:10.3390/ijms241814313)
Supplement: Supplementary file 1 [file ijms-24-14313-s001.zip › ijms-2561962-supplementary.pdf]

Supporting information for

Degradation of Diclofenac by Loaded Solid Superbase-Activated Persulfate

Jiaqi Shi [1,2], Lei Wang [1], Shang Gao [1], Jianbo Huang [1], Hao Yang [1,3], Hao Lu [1,4] and Shaohua Cao [1,*]

1 State Environmental Protection Key Laboratory of Soil Environmental Management and Pollution Control, Nanjing Institute of Environmental Sciences, Ministry of Ecology and Environment of China, Nanjing 210042, China; sjq@nies.org (J.S.); leiwang@nies.org (L.W.); gaoshang@nies.org (S.G.); hjb@nies.org (J.H.); 15215658304@163.com (H.L.)
2 State Key Laboratory of Pollution Control and Resource Reuse, School of the Environment, Nanjing University, Nanjing 210023, China
3 College of Environment, Hohai University, Nanjing 210098, China
4 College of Resources and Environmental Engineering, Hefei University of Technology, Hefei 230009, China
* Correspondence: csh@nies.org; Tel.: +86-02558965825

S1. Characterisation of materials

S1.1 SEM

The scanning electron microscope (SEM) photographs were taken on an S-4800 (Hitach) with an accelerating voltage of 3~5 kV and magnification of 10.00 KX. The working distances were 5.8 mm and 4.6 mm respectively, and the pixel resolution used in the imaging was 1 μm.

The SEM of KAl, (26)KSM(10), γ-Al_2O_3 and SBA-15 are shown in Figure S1. Compared Figure S1(a) with Figure S1(c), the load of KNO_3 made the surface morphology of γ-Al_2O_3 greatly changed. Small pores were formed with the surface of the particles becoming rough, and the particle size was increased. Similarly, comparing Figure S1(b) with Figure S1(d), it could be seen that the surface of (26)KSM(10) was much more porous than SBA-15, but the intensity of the pores were less than KAl, indicating that MgO reduces the damage of KNO_3 to SBA-15 structure to some extent.

S1.2 BET analysis

The N_2 adsorption-desorption isotherms of KAl and (26)KSM(10) are shown in Figure S2(a) and Figure S2(b), respectively. The isotherms are both of type IV with a H3 hysteresis loop based on International Union of Pure and Applied Chemistry (IUPAC) guidelines, indicating that there are a large number of micropores and mesoporous in KAl and (26)KSM(10). The details of the pore characteristics are listed in Table S1. It can be seen that both materials are mesoporous. The (26)KSM(10) had much bigger surface area and pore volume than KAl, but had a slightly smaller average pore diameter. Figure S2(c) shows the pore size distribution curves, indicating that KAl has mesoporous between 8–36 nm, while (26)KSM(10) has a small number of micropores between 1–2 nm and lots of mesoporous between 4–36 nm.

S1.3 CO_2-TPD analysis

Figure S3 shows CO_2-TPD spectra of KAl and (26)KSM(10). It can be seen that KAl has two obvious desorption peaks at 189°C and 420°C respectively. The CO_2 desorption peak in the low temperature zone below 200°C indicates that the material

itself has weak alkalinity, while the obvious CO_2 desorption peak in the high temperature zone between 200 ºC ~ 450ºC indicates that KAl has strong alkalinity [1, 2]. Similarly, two peaks at appeared in (26)KSM(10). The peaks temperatures were 155°C and 383°C, respectively, and the (26)KSM(10) has more quantity of strong alkaline sites compared with KAl indicated by the larger peak area.

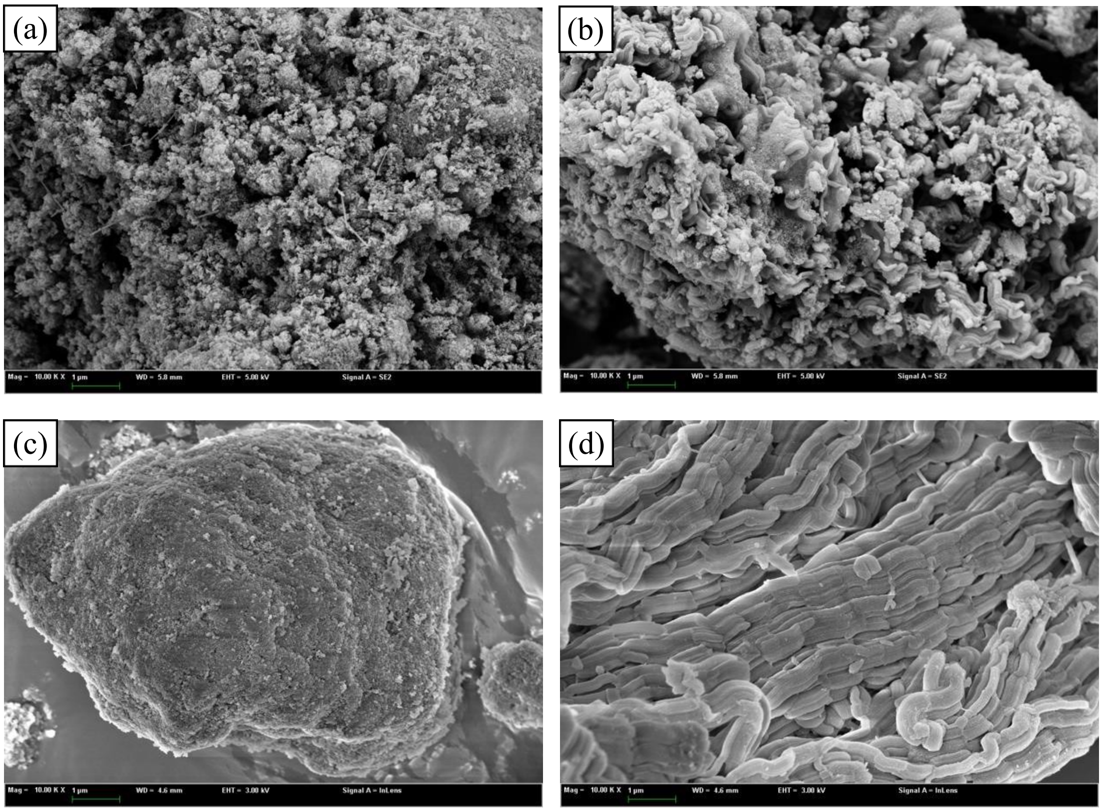

Figure S1 Electron microscopy characterizations of (a) KAl, (b) (26)KSM(10), (c)γ-Al_2O_3, (d) SBA-15

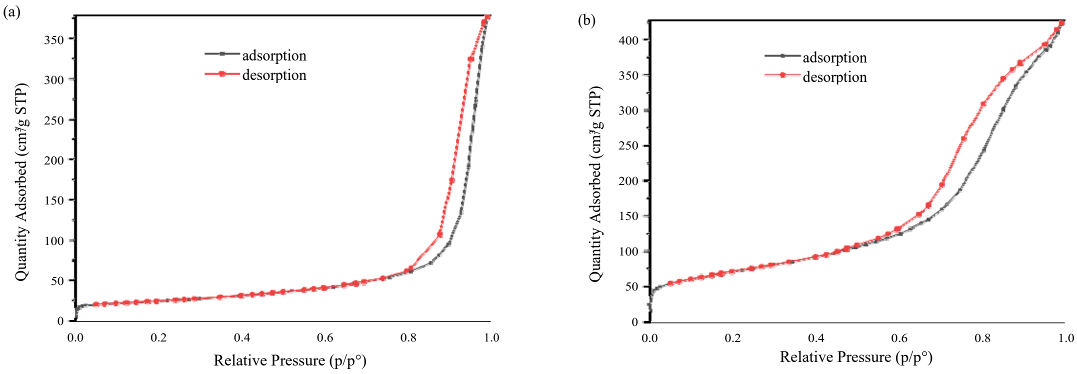

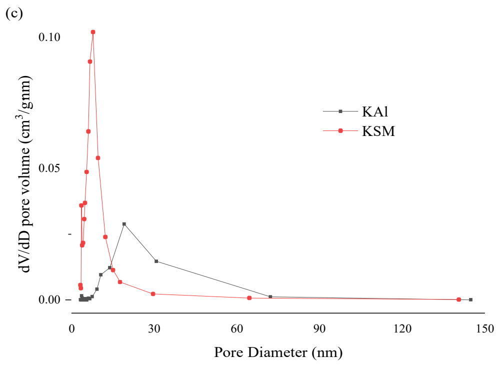

Figure S2 N_2 **adsorption-desorption isotherms of (a) KAl and (b) (26)KSM(10) and (c) pore size distributions calculated by the Barrett-Joyner-Halenda (BJH) method.**

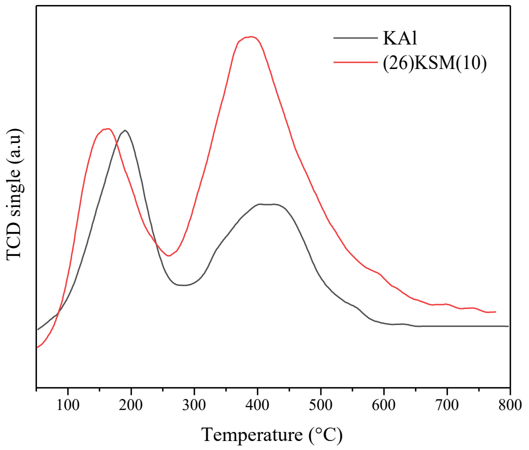

Figure S3 CO_2**-TPD profile of the superbases**

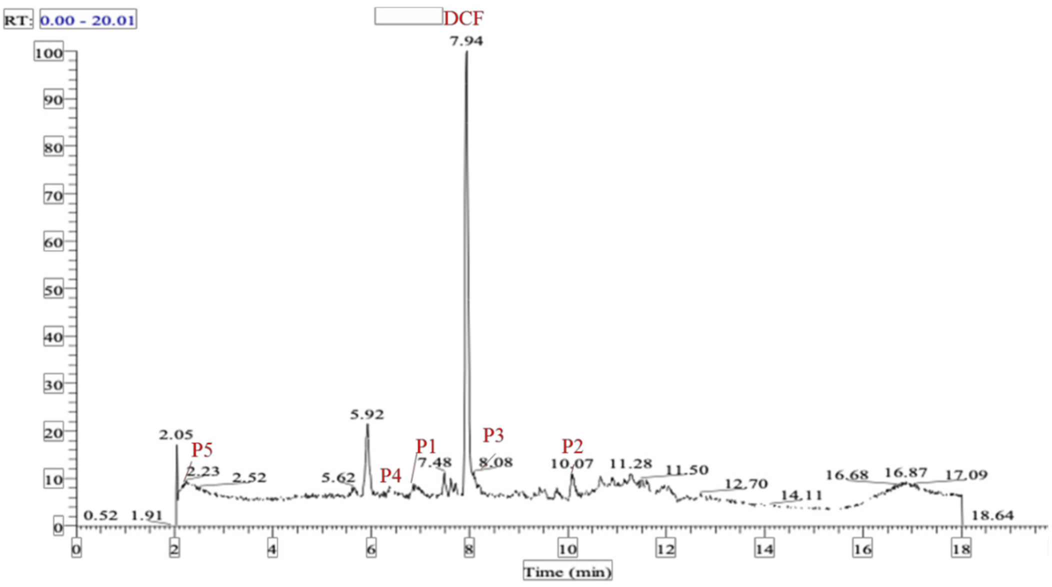

Figure S4 **The chromatogram of DCF and oxidation products**

Table S1 Sample surface area, pore volume and average pore diameter

Sample	BET surface area ($m^2 \cdot g^{-1}$)	Mesoporous surface area ($m^2 \cdot g^{-1}$)	Total pore volume ($cm^3 \cdot g^{-1}$)	Mesoporous volume ($cm^3 \cdot g^{-1}$)	Average pore diameter (nm)
KAl	83.82	78.94	0.5818	0.5768	27.76
(26)KSM(10)	243.5	237.0	0.6560	0.6387	10.78

Reference

[1] Y. Wang, W.Y. Huang, Y. Chun, J.R. Xia, J.H. Zhu, Dispersion of potassium nitrate and the resulting strong basicity on zirconia, Chem. Mat. 13 2001 670-677. https://doi.org/10.1021/cm000213n.

[2] S. Yin, B. Xu, S. Wang, C. Au, Nanosized Ru on high-surface-area superbasic ZrO2-KOH for efficient generation of hydrogen via ammonia decomposition, Applied Catalysis A: General. 301 2006 202-210.https://doi.org/10.1016/j.apcata.2005.12.005.
